# The mechanistic and evolutionary aspects of the 2′- and 3′-OH paradigm in biosynthetic machinery

**DOI:** 10.1186/1745-6150-8-17

**Published:** 2013-07-08

**Authors:** Mark Safro, Liron Klipcan

**Affiliations:** 1 Department of Structural Biology, Weizmann Institute of Science, 234 Herzl Street, Rehovot 76100, Israel; 2 Medical Research Council, Mitochondrial Biology Unit, Wellcome Trust / MRC Building Hills Road, Cambridge CB2 0XY, United Kingdom

**Keywords:** Aminoacyl-tRNA synthetases, Elongation factor EF-Tu, Ribosome, 2′-3′ hydroxyls of the ribose

## Abstract

**Background:**

The translation machinery underlies a multitude of biological processes within the cell. The design and implementation of the modern translation apparatus on even the simplest course of action is extremely complex, and involves different RNA and protein factors. According to the “RNA world” idea, the critical link in the translation machinery may be assigned to an adaptor tRNA molecule. Its exceptional functional and structural characteristics are of primary importance in understanding the evolutionary relationships among all these macromolecular components.

**Presentation of the hypothesis:**

The 2′-3′ hydroxyls of the tRNA A76 constitute chemical groups of critical functional importance, as they are implicated in almost all phases of protein biosynthesis. They contribute to: a) each step of the tRNA aminoacylation reaction catalyzed by aminoacyl-tRNA synthetases (aaRSs); b) the isomerase activity of EF-Tu, involving a mixture of the 2′(3′)- aminoacyl tRNA isomers as substrates, thereby producing the required combination of amino acid and tRNA; and c) peptide bond formation at the peptidyl transferase center (PTC) of the ribosome. We hypothesize that specific functions assigned to the 2′-3′ hydroxyls during peptide bond formation co-evolved, together with two modes of attack on the aminoacyl-adenylate carbonyl typical for two classes of aaRSs, and alongside the isomerase activity of EF-Tu. Protein components of the translational apparatus are universally recognized as being of ancient origin, possibly replacing RNA-based enzymes that may have existed before the last universal common ancestor (LUCA). We believe that a remnant of these processes is still imprinted on the organization of modern-day translation.

**Testing and implications of the hypothesis:**

Earlier publications indicate that it is possible to select ribozymes capable of attaching the aa-AMP moiety to RNA molecules. The scenario described herein would gain general acceptance, if a ribozyme able to activate the amino acid and transfer it onto the terminal ribose of the tRNA, would be found in any life form, or generated *in vitro*. Interestingly, recent studies have demonstrated the plausibility of using metals, likely abandoned under primordial conditions, as biomimetic catalysts of the aminoacylation reaction.

**Reviewers:**

This article was reviewed by Henri Grosjean, Manuel Santos and Eugene Koonin. For complete reviews, go to the Reviewers’ reports section.

## Background

The design and implementation of the modern system of genetic code translation, even at its simplest, is an extremely complex process. The heart of the biosynthetic machinery is the ribosome, ribonucleoprotein providing link amino acids together based on the information encoded in mRNA. The ribosome governs the collective effects of various RNA, protein factors and other essential components to good advantage. Almost all stages of this intricate process are mediated by adaptor tRNAs, which recognize a triplet of nucleotides on the mRNA through a complementarity determining region called an anticodon, and carry a covalently attached cognate amino acid [1].

The attachment of amino acids to specific adaptor tRNAs is mediated by a group of canonical enzymes known as aaRSs, which catalyze a two-step aminoacylation reaction. The selectivity in recognition of both the amino acid to be activated and the cognate tRNA constitutes a crucial step in the fidelity of polypeptide chain formation. Aminoacylated tRNA is delivered to the ribosome in a ternary complex of aminoacyl-tRNA• GTP•EF-Tu (where GTP is guanosine triphosphate and EF-Tu is the elongation factor), which plays a substantial role in the decoding process.

At its core the ribosome might be thought of as an ancient molecular machine governed by RNA. The modern ribosome is a 2.5-MDa riboprotein assembly composed of two unequal subunits that associate upon initiation of the biosynthetic cycle. The mRNA is decoded at the small subunit, whereas peptide bond formation occurs on the large subunit, within the PTC, buried deep inside a cavity and primarily built of ribosomal RNA [2-4]. Two critical issues still remain to be clarified regarding the translation machinery: the control mechanisms underlying both peptide bond formation, and the translocation of the tRNA molecules.

**Figure 1:**
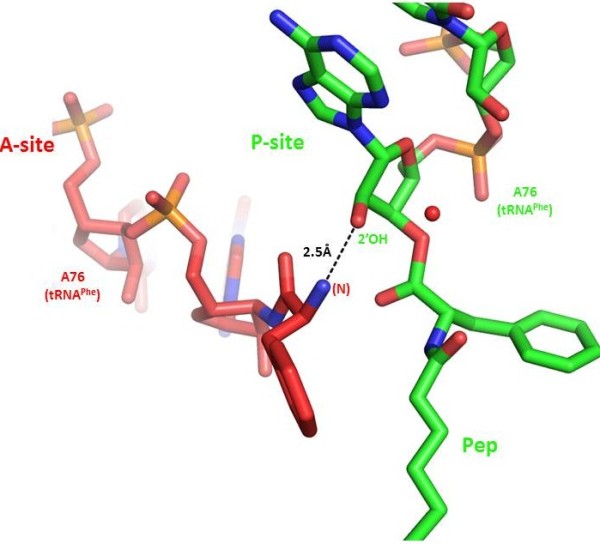
**Model of the peptidyl-transferase reaction. In transition state analogs, the only group found within hydrogen-bonding distance is the 2′-OH of A76 of peptidyl-tRNA in the P site.** PDB Code: 1VQN.

Over the years peptidyl transferase activity saga has undergone dramatic changes [5,6]. Initial models were based on a ribosomal protein-based mechanism [7]. The discovery of RNA-facilitated activity made rRNA-mediated catalysis on the ribosome fashionable. With the availability of high-resolution crystallographic structures of ribosomal subunits isolated from various sources, knowledge of the structural organization and functioning of the organelle took a quantum leap forward, and compellingly confirmed that peptidyl transferase is an RNA enzyme [2,8]. More recent findings revealed that the true catalyst behind peptide bond formation appears to be the aminoacylated tRNA molecule. Furthermore, the remarkable substitution of the tRNA terminal ribose 2′-OH group with deoxy (H) or fluoro (F) 2′-H or 2′-F results in at least a 10^6^-fold reduction in the rate of peptide bond formation [9]. This indicates that the 2′-OH group is a critical component of the reaction (Figure 1).

Based on data from the crystal structures, and biochemical analysis of translational machinery components, we put forward a hypothesis whereby the mechanism of peptide bond formation on the ribosome co-evolved, in parallel with the aminoacylation and editing activities of aaRSs. Meanwhile, the elongation factor co-evolved with some aaRSs, to securely transfer 3′-OH aminoacylated tRNAs on the ribosome.

## Presentation of the hypothesis

### AaRSs and their role in translation

Prior to polypeptide chain synthesis, aaRSs provide the ribosome with amino acid substrates in the form of aminoacyl-tRNAs (aa-tRNAs). AaRSs constitute a large family of enzymes partitioned into two distinct classes varying greatly in their structural organization, and their mode of interaction with tRNA [10]. Class I aaRSs exhibit catalytic domains containing the classical nucleotide-binding Rossmann fold, and amino acylation reactions involve Trp, Tyr, Gln, Glu, Lys-1, Val, Ile, Leu, Met, Arg and Cys substrate amino acids. Class II aaRSs exhibit catalytic domains built around an antiparallel β-sheet flanked by α-helices, and acylate their cognate tRNA with Pro, Thr, Ser, Asp, Asn, Lys-2, His, Ala, Gly, Phosphoserine, Pyrrolysine, and Phe substrate amino acids. Along with amino acids delivering to the ribosome, aaRSs maintain the high fidelity of genetic code translation. They catalyze the attachment of amino acids to the 3′- end of the tRNA in a two-step aminoacylation reaction: a) first, the aaRS binds ATP and the amino acid (aa), catalyzing formation of the intermediate aminoacyl-adenylate (aa-AMP); and b) secondly, the amino acid is transferred to the cognate tRNA, covalently binding to either the 2′OH or 3′OH of the A76 terminal ribose. For class I aaRSs, the primary site of aminoacylation is the 2′-OH group of ribose, whereas for class II aaRSs, this site is the 3′-OH group (with the exception of PheRS, which structurally belongs to class II aaRSs, but aminoacylate the 2′-OH group) [11].

**Figure 2:**
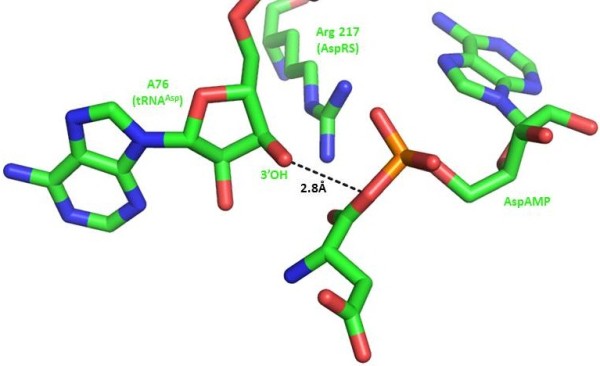
**The second step of the aminoacylation reaction catalyzed by AspRS.** The terminal 3′-OH ribose is positioned for attack on the aspartyl-adenylate carboxyl group. The positive-charged and class II invariant arginine contributes to the neutralization and stabilization of the aspartyl-adenylate phosphate group. PDB Code: 1C0A.

According to the well-studied mechanism of amino acid activation, the geometry and charge of a pentacovalent transition state should be stabilized by positively charged amino acids and divalent cations (magnesium or possibly manganese) [12]. A large body of evidence [2,3,13] suggests that upon transition state formation, ATP and amino acid substrates are optimally oriented for an “in-line” displacement reaction to occur. Analogous to the formation of peptidyl-tRNA on the ribosome, synthesis of aminoacyl-tRNA does not requires direct involvement of aaRS residues in the catalytic process (Figure 2) [14].

The critical role played by aaRSs in the evolutionary history of the translational apparatus, and in the maintenance of fidelity in genetic code translation, enables one to hypothesize that aaRSs were among the earliest proteins to appear: at present, they exist in all living organisms [15-17]. Moreover, one may speculate that class II aaRSs, as well as the amino acids associated with them, predate those of class I [16]. Firstly, class II aaRSs predominantly aminoacylate tRNA with simpler, smaller amino acids (e.g., Gly, Pro, Ala, Asp, Thr and Ser), many of which play crucial roles in the formation of protein folds. Notably, these same amino acids were found to be the most abundant in the classical Miller experiments, which attempted to imitate conditions of primordial environments. Our second argument follows from the observation that biosynthetic pathways of class II-associated amino acids usually involve fewer intermediate stages, as compared to class I representatives; furthermore, they are mostly synthesized from components of glycolysis and the Krebs cycle pathways [16]. Furthermore, many class II amino acids e.g. Ala, Asp, Gly, Ser, and Thr (all 3′-OH aminoacylated) serve as metabolic precursors for all the other amino acids [16]. Thirdly, two-(G and C) and three-letter-based codons (G, C and A) are believed to be related to ancient codons [18]. Analysis of these codon triplets revealed that most often, they associate with class II amino-acids and with their partner aaRSs. Detailed investigations showed that nine out of the 12 amino acids codified by these codons belong to class II-type amino acids, aminoacylating the 3′-OH of cognate tRNA [16].

### The elongation factor EF-Tu as a 2′OH-3′OH isomerase

During the elongation step of protein synthesis, aminoacylated tRNA is delivered to the ribosomal PTC, aided by the GTP-binding elongation factor EF-Tu. The GTP-bound form of EF-Tu brings the new aa-tRNA to the empty A-site of the ribosome, in order for decoding to occur. This triggers GTP hydrolysis, and leads to the release of GDP-bound EF-Tu from the ribosome. The initial step of EF-Tu-GTP-aa-tRNA interaction with the ribosome, known as the “encounter complex”, entails codon-independent binding. The rate of binding is determined by EF-Tu interactions with the ribosome: this step proceeds much more slowly with aa-tRNA alone [19]. The rate constant for complex formation at 20°C is about 10^-8^ M^-1^ s^-1^[18,20]; relatively high, as compared to typical rate constants previously reported for other macromolecular systems. This indicates that the macromolecular association of the ternary complex with the ribosome is not entirely random. On the other hand, the fact that proper binding of aminoacylated tRNA to the ribosome may happen without EF-Tu involvement, suggests that this primitive translation system is also active, even in the absence of protein factors [21,22].

A secondary function of EF-Tu is worthy of mention, and crucial in determining the finer details of the modern translational apparatus. EF-Tu also acts as an isomerase, utilizing a mixture of the 2′(3′)- aminoacyl tRNA isomers as a substrate, and converting them to uniform 3′-complexes [11]. Both 2′- and 3′-aminoacyl isomers of ‘deoxy’ RNAs can bind EF-Tu · GTP, albeit with 50-fold lower efficiency as compared to the native 2′(3′)-aminoacyl-tRNA [23,24]. The preference for the 3′-isomer of aa-tRNAs, upon complex formation with EF-Tu, was also demonstrated by NMR results published by S. Yokoyama et al. [25], and supported more recently by X-ray analysis of EF-Tu complexed with aa-tRNA [26], and data from steady-state and transient kinetic experiments [26,27]. Under standard physiological conditions, the transacylation rate from 2′-OH to 3′-OH isomers is slower than the overall rate of polypeptide chain elongation in the ribosome. Taken together, these findings suggest that the appearance of aaRSs catalyzing 2′-OH aminoacylation should be accompanied by the appearance of Ef-Tu stabilizing 3-OH aminoacylated isomers.

### The co-evolution of Ef-Tu and class I aaRSs

Structural and phylogenetic analyses suggest that class I aaRSs evolved relatively late in evolution, from enzymes involved in cofactor biosynthesis [22]. Class I aaRSs were originally adapted to aminoacylate tRNA, for the most part with the “large” biosynthetically complex amino acids. Thus, it seems likely that class I aaRSs evolved in an environment populated by a number of proteins, coupled with the relatively efficient translation machinery. It has now become clear that the kinetic mechanism of aminoacylation differs between the two classes, and that they do not, as previously proposed, “represent common orthogonal chemical solutions to the aminoacylation of the tRNA” [28]. Many class I enzymes (e.g., CysRS, ValRS, IleRS, ArgRS, and GlnRS) exhibit burst kinetics, suggesting that product release is rate-limiting; whereas class II enzymes are rate-limited at the amino acid activation stage [29]. Moreover, aminoacylation reactions performed in the presence of EF-Tu demonstrate that the elongation factor selectively enhances the activity of class I aaRSs by promoting enzyme turnover.

**Figure 3:**
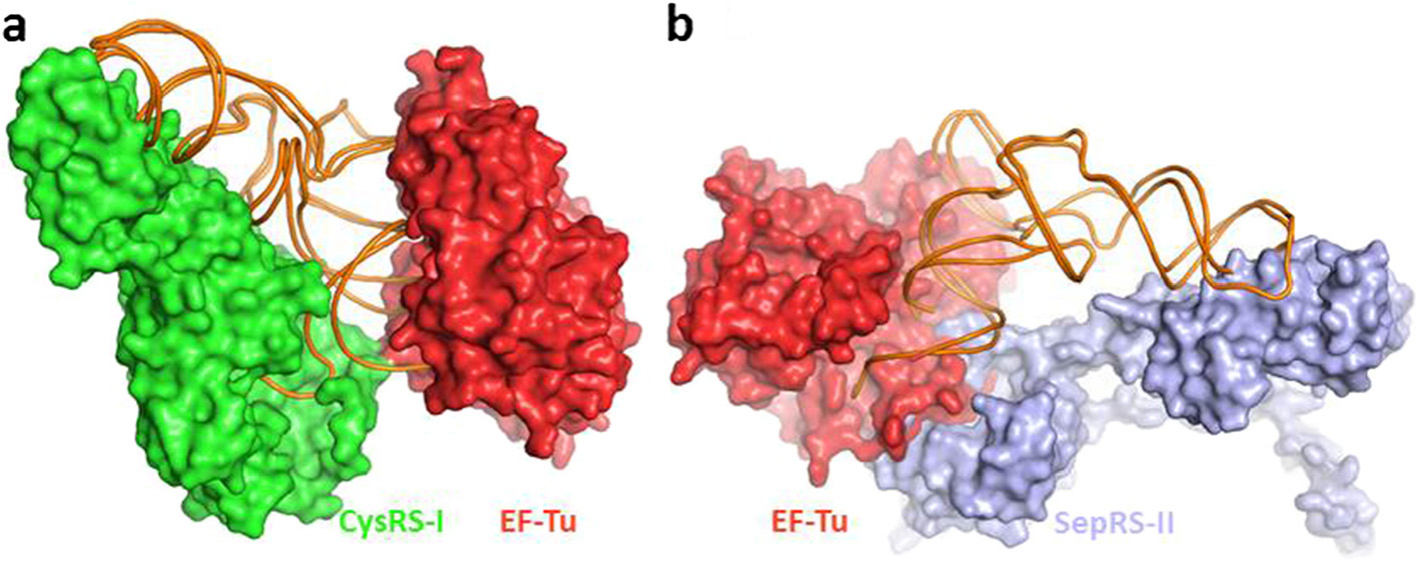
**Superposition of crystal structures for two class I and class II aaRSs complexed with tRNAs, on that of EF-Tu and tRNA. (a)** Overlay of class I CysRS and EF-Tu with their tRNAs (CysRS in green, PDB Code-1V0B; EF-Tu in red, PDB Code 1B23; tRNAs in orange). **(b)** Overlay of class II SepRS (PDB Code: 2DU3) and EF-Tu complexed with their tRNAs (SepRS in blue; EF-TU in red; tRNAs in orange). In view of the fact that the structural folds of tRNAs in all complexes are similar in appearance, the models were generated by superposition of the respective tRNA molecules.

In essence, formation of the EF-Tu ternary complex helps to activate the release of aa-tRNAs from class I aaRSs, thereby speeding up the process of protein synthesis. However, formation of the ternary complex aaRS−EF-Tu−tRNA occurs in class I aaRSs only (Figure 3a). EF-Tu fails to exhibit significant binding affinity to class II aaRSs, as they basically attach amino acids to the A76 3′-OH, thereby forming, in solution, more stable 3′-isomers, already prepared for delivery to the ribosome. Moreover, class II aaRSs approach the tRNA acceptor stem from the same side of the major groove as EF-Tu. Thus, formation of a stable assembly involving class II aaRSs, EF-Tu, and aa-tRNA is hardly probable, in view of its sterical unfeasibility (Figure 3b).

Transformation of the tRNA aminoacylated at the 2′-OH of the terminal ribose to the 3′-OH variant, and utilization of the EF-Tu in securing this state, underscores the fundamental role played by tRNA aminoacylated specifically on the 3′-OH. Involvement of the additional enzyme converting 2′-OH to the 3′-OH aminoacylated tRNA and, at a later stage, delivering the stabilized variant to the PTC of the ribosome supporting the hypothesis that class I aaRSs evolutionarily evolved only after the appearance of EF-Tu.

### The transition from 3′-OH to 2′-OH aminoacylation

Many methanogenic Archaea lack a gene encoding canonical class cysteinyl-tRNA synthetase (CysRS), as well as genes analogous to those involved in bacterial or eukaryotic cysteine biosynthesis [30,31]. However, an indirect pathway to the formation of Cys-tRNA^Cys^ in these organisms was recently revealed [30]. In the first stage, the enzyme *O*-phosphoseryl-tRNA synthetase (SepRS) ligates O-phosphoserine to tRNA^Cys^; subsequently, *O*-phosphoseryl-tRNA^Cys^ is converted to Cys-tRNA^Cys^ by Sep-tRNA, forming Cys-tRNA synthase (SepCysC). Such reactions can therefore be classified as tRNA-dependent amino acid biosynthesis. It is conceivable that the tRNA-dependent pathway of amino acid biosynthesis represents a remnant of RNA-based metabolism [32]. The class II enzyme SepRS most likely utilizes the 3′-OH isomer of tRNA as a substrate. Ancient organisms that incorporated cysteine into polypeptide chains took advantage of this pathway, while in the modern world, the majority of organisms utilize a standard pathway involving class I CysRS.

The close association between the tRNA-dependent pathways of cysteine aminoacylation in ancient Archaea and in modern organisms provides a good example of 3′-OH to 2′-OH directional transition during evolution. First, both CysRS and SepRS recognize similar identity elements on the tRNA^Cys^ molecule [33]. Secondly, bacterial CysRS is able to aminoacylate both the 2′-OH and 3′-OH groups of tRNA^Cys^ (reviewed in [11]). *In vitro*, the reaction rate of 2′-OH aminoacylation is about one order of magnitude faster than that of 3′-OH acylation [29]. The EF-Tu ternary complex tightly binds CysRS in the nano-molar range, and appears to enhance the stabilization of 3′-OH as a preferred site for aminoacylation reactions [29].

**Figure 4:**
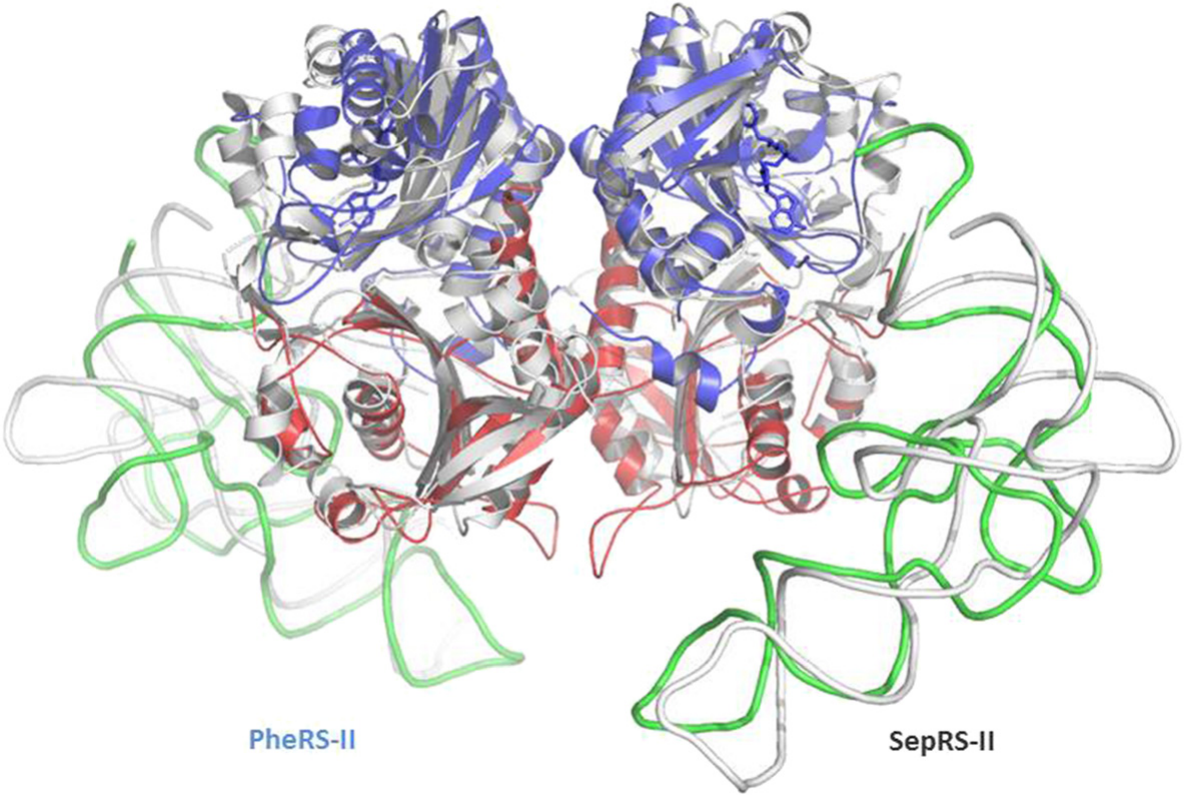
**Superposition of the catalytic cores of class II SepRS and PheRS (PheRS colored in red, blue, and green.** SepRS colored in white).

**Figure 5:**
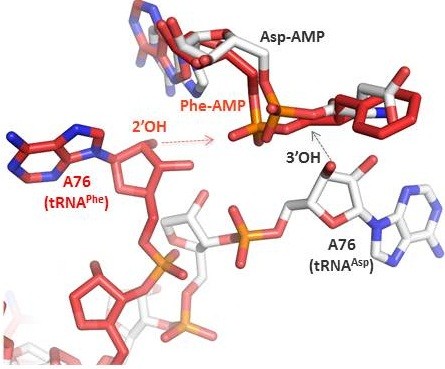
**Comparison of the second step of the reaction catalyzed by PheRS (red) and AspRS (white).** In contrast to other class II aaRSs, in PheRS, the 2′-OH ribose is most suitable for attack on the phenyl-adenylate carboxyl group.

A striking structural similarity is seen when the central part of the *M. maripaludis* SepRS tetramer is superimposed onto the (αβ)_2_ heterotetramer of *T. thermophilus* PheRS: the r.m.s.d. between 525 corresponding C_α_ atoms in the two assemblies is only 2.2 Å [34-38]. Four catalytic domains of the tetrameric SepRS are congruent to the catalytic and catalytic-like domains of *T. thermophilus* PheRS pertaining to α- and β-subunits, respectively (Figure 4). In both proteins, the core region is formed by a four-helix bundle interface [39]. The similarity of the two enzymes in their quaternary organization is of particular interest, since the divergence of the two families may predate the LUCA. Notwithstanding the fact that both enzymes share a common origin, and structurally belong to class II aaRSs, PheRS, in contrast to SepRS, attaches an amino acid to a 2′-OH of terminal adenosine. None of the crystal structures of PheRS complexed with functional ligands or their analogues revealed a conformation of the CCA end that was appropriate for aminoacylation of the 3′-OH group. This is due to the fact that A76 doesn’t penetrate deeply enough into the active site to create the required distance between the 3’-OH and the α-carbonyl carbon of the PheAMP, suitable for nucleophilic attack (Figure 5). Rather, it is dictated by a trajectory whereby PheRS approaches tRNA^Phe^. Interestingly, mitochondrial PheRS (mitPheRS), as well as cytoplasmic PheRS, utilize the 2′-OH of tRNA^Phe^ as the initial aminoacylation site. However, measurements of the kinetic parameters of tRNA^Phe^-CC-3′dA for mitPheRS revealed that the mitochondrial enzyme requires both hydroxyl groups of the terminal adenosine for efficient aminoacylation to occur [40].

There are solid grounds for believing that PheRS was one of the last class II enzymes to evolve. First, phenylalanine is a biosynthetically complex amino acid: since LUCA, a clear tendency toward increased phenylalanine content in proteins has been seen. Secondly, the crystal structures of bacterial PheRSs, composed of 22 structural domains, have shown that it is the most complex aaRS. Bacterial, archaeal and eukaryotic (cytoplasmic and mitochondrial) PheRSs share a common catalytic fold, with some redesign of the active site residues. The subunit organization of the enzymes, however, differs substantially: loss of certain structural domains, including those involved in anticodon recognition, or acquisition of new ones suggests the emergence of the different binding modes with cognate tRNAs, whose identity elements vary in bacteria and eukaryotes [41,42]. Taken together, these findings suggest that the stereochemical aspects of phenylalanine-specific recognition, and the mechanism of its subsequent activation, appeared prior to the acquisition of aminoacylation by the phe-system. Subsequently, the phe-system continued to evolve beyond the divergence of the primary kingdoms and translational apparatus.

### The editing activity of aaRSs and Ef-Tu

A cornerstone in the evolution of the genetic code entails the transition from early proteins that may have been inherently statistical that is, one of several stereochemically analogous amino acids might have been brought to a given position within the polypeptide chain, to protein with unique sequence and 3D-structure. This transition was basically secured by aaRSs, which guaranteed a selection of proper amino acids bound to cognate tRNAs. The difficulties in discriminating between certain amino acids whose chemical structures bore a close resemblance were finally resolved with the discovery of editing activity that prevents misactivation of non-cognate amino acids (pre-transfer editing) [43] or misaminoacylation of tRNA (post-transfer editing) [44]. The existence of this additional activity appears to maintain the rate of misincorporation of non-cognate amino acids *in vivo*, at the level of approximately one error out of every 10^4^–10^5^ reactions.

It would seem that the proofreading activity of aaRSs evolved early in the evolution of the translation apparatus, as ancient class II aaRSs such as AlaRS, ProRS, ThRS and PheRS acquired editing domains possessing different architectures. As dissociation of tRNAs from enzymes is not a rate-limited state for class II aaRSs, it is feasible that class II aminoacylated tRNA frequently utilizes the mechanism of trans editing. Indeed, as has been demonstrated for AlaRS, recognition of misacylated Ser-tRNA^Ala^ by distinct structural domains in the course of the editing reaction suggests the immediate dissociation of aminoacylated tRNAs from aaRSs and their subsequent rebinding, using different structural modules [45]. In a number of experiments, it was also shown that PheRS utilizes cis- as well as trans-editing mechanisms [46-48]. Moreover, in the presence of EF-Tu, PheRS efficiently competes with the elongation factor to rebind the mischarged Tyr-tRNA^Phe^, and then rapidly hydrolyzes it at the editing site, utilizing the trans- pathway. According to experimental evidence [46], aminoacylation of 2′-OH by PheRS in the presence of EF-Tu is accompanied by stabilization of the 3′-OH isomer. Thus, for PheRS, we anticipate that misacylated tRNA approaches the editing site in both cis- and trans-states, being aminoacylated on the 2′-OH and 3′-OH isomers, respectively. Paradoxically, the same editing site and mechanism of hydrolysis serve for two stereochemically different complexes of substrate amino acids bound at 2′-OH-tRNA (cis-editing) and at 3′-OH-tRNA (trans-editing).

The editing activities of IleRS, LeuRS and ValRS, which together form subclass Ia aaRSs, are known to be performed by the hydrolytic CP1 domain fused to a catalytic core comprising a Rossmann-fold domain. Experimental evidence for the existence of an editing site far removed from the synthetic site was first reported for IleRS [49]. IleRS, LeuRS and ValRS, whose structures are closely related, also display variations in their hydrolytic mechanisms. IleRS edits the 3′-OH acylated tRNA, and therefore most likely requires EF-Tu for stabilization of the 3′-OH isomer [50]; while LeuRS and ValRS, which edit tRNAs specifically from 2′-OH acylated tRNA, probably utilize the cis mechanism of editing [50,51].

It might be well to point out that IleRS and LeuRS also utilize a distinctive tRNA-dependent pre-transfer editing activity within their synthetic sites [50,51].

## Testing and implication of the hypothesis

Systematic analyses of structural and kinetic data revealed that the principal components of the translation apparatus, such as aaRSs, and the PTC of the ribosome, only assist in the proper positioning of the reactants, and do not directly participate in catalytic reactions. It is remarkable that, in both the synthetic active site of aaRSs and in the PTC, the major role is allocated to substrate tRNA molecules. We therefore propose that even in the modern biosynthetic machinery, the tRNA molecule functions merely as a ribozyme. In fact, some semblance of a common strategy may be revealed between ribozymes catalyzing the phosphophoryl-transfer reaction, and peptide bond formation catalyzed by tRNA. For example, the 2′-OH in group II introns and the terminal 3′-OH in group I introns, are both directly involved in the intron cleavage reaction (reviewed in [52]).

The two vicinal hydroxyl functional groups are known to have been present in the A76 ribose before the partitioning of aaRSs into two “orthogonal” chemical solutions to the aminoacylation of the 3′ end of the tRNA occurred. Although 3′-aminoacyl-adenosine and 3′-aminoacyl-tRNA are more favoured energetically as compared to the corresponding 2′-isomers [53], aaRSs demonstrate roughly comparable numbers of 2′- and 3′- aminoacylation sites in the two aaRSs classes. Attachment of amino acids to 2′-OH or 3′-OH isomers was recently shown by means of biomimetic systems; a preference for 3′-OH aminoacylation was also observed [54]. It seems that the prevalence of the ribose 3′-OH bound polymer is a universal trait, inherited due to chemical necessity. It is also implied in the retention of 2′- and 3′-specificity in aaRSs belonging to prokaryotes and eukaryotes. In particular, it should be emphasized that this principle holds true, even with the distinctions in tRNA binding modes and topology of the aaRS active sites observed among the three kingdoms. From this, it immediately follows that aaRS side chains forming interior of the synthetic and editing sites are closely implicated in the selection of 2′- or 3′-OH variants. Thus, the architecture and domain composition of aaRSs throughout evolution evolved at least synchronously with their ability to discriminate between 2′ and 3′ OH-groups in aminoacylation reactions, or later on. The chemical activity of only two 2′-3′ OH-groups at the 3′-terminal adenosine of tRNA, coupled with evolutionary pressure for new functions of aaRSs, EF-Tu and ribosomes, created a great deal of mechanistic diversity and specificity, on the way to formation of newly synthesized polypeptide chains.

## Reviewer’s comments

Reviewer 1: Dr. Henri Grosjean (nominated by Dr. P. Lopez-Garcia)

In this manuscript, the authors hypothesize that the specific functions assigned to the 2′-3′ hydroxyls during peptide bond formation on the ribosome has co-evolved with the mechanism of tRNA aminoacylation and editing activities by the 2 classes of aminoacyl-tRNA synthetases (aaRSs), and with the isomerase activity of EF-Tu employing a mixture of the 2′(3′)-aminoacyl-tRNA isomers as substrates. Their main idea of the paper is that the true catalysts of peptide bond formation on the ribosome are the aminoacylated tRNA molecules, the aaRSs and the PTC of ribosome mainly allowing the proper positioning of the aa-tRNAs with the respective active sites. In other words, the tRNA molecule merely functions as a ribozyme, an idea that fits with the now generally admitted hypothesis that ancient protein synthesis machinery was governed by RNA. This paper is a logical prolongation of an earlier work (J. Theor Biol-2004) more focused on the coevolution hypothesis between amino acid biogenesis, aaRSs and the genetic code. These two papers complete each other very well. The present manuscript is clearly written, and while no experimental evidences are provided, the hypothesis is convincing.

Minor remarks: Page 8 GCA ancient codon? This should be developing a bit because the argument is not obvious. Page 9 > > 20° should be 20°C of course. Page 15, cys-editing should be written cis-editing (this mistake occurs 3 times in the text).

Authors’ response: We accepted all the remarks of Reviewer 1, and corrected the text accordingly. As to the antiquity of the GCA codons, we added a few sentences at the end of that discussion, to clarify our arguments.

Reviewer 2: Prof. Manuel Santos (nominated by Prof. Yitzhak Pilpel)

In this hypothesis paper Mark Safro and Liron Klipcan put forward the hypothesis that the functions of the 2′and 3′ hydroxyls of the conserved tRNA 3′-terminal adenosine (A76) observed during peptide bond formation at the peptidyl transferase centre (PTC) of the ribosome co-evolved with the two modes of attack on the aminoacyl-adenylate carbonyl typical of the two classes of aaRSs and also with the isomerase activity of EF-Tu. The authors recognize the fundamental roles of the 3′-OH and 2′-OH groups in protein synthesis and develop their arguments with several examples that highlight the relevance of the 2′-OH and 3′-OH chemistry at the amino acid activation, editing, transfer to the elongation factor and PTC levels. This is an expected outcome of the 2′-OH and 3′-OH multilayered chemistry, it would be surprising if it worked in a different manner. The value of this highly descriptive hypothesis paper that reviews old concepts is that it highlights the overlooked chemistry of the 2′-OH and 3′-OH, bringing it to current debate. This is important and sufficient to recommend its publication. However, there are several aspects of the paper that should be improved before publication, namely:

1. The first part of the manuscript (including the abstract) is poorly written and should be improved. There are many grammatical errors along the manuscript.

2. On page-4, third paragraph, the authors state that “the ribosome is a spectacular fossil artefact”. Looking at the ribosome as a fossil artifact is simply wrong. This should be changed to make to sentence clear.

3. The 3 subheadings dedicated to the aminoacylation reaction, the aaRS classes and the early role of Class-II aaRS in genetic code translation should be fused into a single subheading and the text shortened. It is too descriptive and repetitive.

4. Page-14, second paragraph. The sentence “….transtion from statistically encoded proteins to error-prone translation” should be clarified. It does not make sense.

5. Page -14, third paragraph, additional references should be included. This is a highly speculative paragraph that could be improved.

Quality of written English: Needs some language corrections before being published.

Authors’ response: As to the criticism of the sentences on pages 4 and 14 we formulated them in more clear form by modifying the text thus: a) the sentence on page 4 now reads, “The ribosome is an ancient molecular machine governed by RNA”; b) the corrected sentence on page 14 now reads, “A cornerstone in the evolution of the genetic code entails the transition from early proteins that may have been inherently statistical; that is, one of several stereochemically analogous amino acids might have been brought to a given position in the polypeptide chain, to protein with unique sequences and 3D-structures.” According to the referee’s request we added references within the third paragraph on page 14 and 15.

The authors agreed with the referee’s suggestion to improve the first part of the manuscript by combining three subheadings of the manuscript dedicated to the aminoacylation reaction, the aaRS classes, and the early role of Class-II aaRS in genetic code translation, into a single section. We fully rewrote this part of the manuscript. While we agree with the reviewer that the introduction should contain a more comprehensive description of the translational apparatus, we would note that the modern system of genetic code translation, even at its simplest, is an extremely complex process, and includes a fair number of macromolecular components. A detailed description of even its major elements would increase the length of the manuscript dramatically.

Reviewer 3: Dr. Koonin E.V.

This article discusses a problem that is both enormously important and staggeringly hard, namely the origin of the modern-type translation system. The authors suggest propose several interesting ideas and make remarkable inferences. To me, the most striking point is that tRNAs effectively act as ribozymes in the modern translation system, both in the aminoacylation and in the peptidyltransferase reactions. Even if not entirely new, this is a startling conclusion, and I find it regrettable that the authors explicitly mention it only in passing, in the Concluding Remarks. The main point of the article, however, is the hypothesis that modern-type translation started with aminoacylation of 3′-OH of A76 in tRNAs that is facilitated by Class II aaRS. Subsequently, under this scenario, Class I aaRS that initially aminoacylate at 2′-OH of A76, followed by isomerization facilitated by EF-Tu, were added to the system. The idea that 3′-OH aminoacylation is primordial certainly is plausible, given that this is the final form in which aminoacyl-tRNAs participate in peptide synthesis. Furthermore, the association of Class II aaRS with chemically simple and supposedly primordial amino acids is compatible with the primacy of this class. However, the evidence of this primacy from protein sequence and structure comparison is weak at the very best. There is just as good a reason to believe that Class II aaRS evolved from coenzyme biosynthesis enzymes (biotin synthetase, same superfamily in SCOP) as there is for class I aaRS, it is just less well publicized. Furthermore, the fact that the substrate-binding domain of class I aaRS belongs to one of the most common, simple protein folds (the Rossmann fold) whereas Class II enzymes adopt a more rare and complex fold, might be used as argument for the primacy of Class I. Moreover, the author repeatedly imply that the transition from statistical peptide synthesis to modern type translation was brought about by the recruitment and diversification of the aaRS. This is extremely unlikely to be the case because both classes of the aaRSs seem to be rather late arrivals in the evolution of the respective protein folds, their emergence being antedated by extensive protein evolution [1,2]. The same holds for EF-Tu which belongs to a small branch within the huge GTPase superfamily [3]. Thus, counterintuitive as that might seem, apparently, there was a high-fidelity RNA-based translation system, and one would think that the roots of the key features of modern translation should be sought there. In that regard, it is very strange that the authors do not examine the properties of the small aminoacylating ribozymes which are among the most remarkable molecules that are relevant for the origin of translation. The GUGGC-3′ ribozyme indeed aminoacylates at 3′-OH [4] which is fully compatible with the primacy of this reaction.

Thus, the hypothesis proposed by the authors in itself is indeed plausible and deserves publication and discussion. However, in the present manuscript, much of the argument is missing, flawed, muddled or tangentially relevant. In addition to the major issues outlined above, I find the discussion of cysteine incorporation in Archaea (which the authors consider to be ancestral without any good reason) as well as the mechanism of PheRS to be largely irrelevant and more confusing than enlightening.

1. Aravind L, Mazumder R, Vasudevan S, Koonin EV: **Trends in protein evolution inferred from sequence and structure analysis. ***Curr Opin Struct Biol.* 2002, **12**:392-399.

2. Aravind L, Anantharaman V, Koonin EV: **Monophyly of class I aminoacyl tRNA synthetase, USPA, ETFP, photolyase, and PP-ATPase nucleotide-binding domains: implications for protein evolution in the RNA**. *Proteins* 2002, **48**:1-14.

3. Leipe DD, Wolf YI, Koonin EV, Aravind L: **Classification and evolution of P-loop GTPases and related ATPases**. J Mol Biol 2002, 317(1):41-72.

4. Yarus M: **The meaning of a minuscule ribozyme**. *Philos Trans R Soc Lond B Biol Sci.* 2011, **366**:2902-2909.

Authors’ response:

a. Reviewer’s comment: “..the association of Class II aaRS with chemically simple and supposedly primordial amino acids is compatible with the primacy of this class. However, the evidence of this primacy from protein sequence and structure comparison is weak at the very best. There is just as good a reason to believe that Class II aaRS evolved from coenzyme biosynthesis enzymes (biotin synthetase, same superfamily in SCOP) as there is for Class I aaRS, it is just less well publicized. Furthermore, the fact that the substrate-binding domain of Class I aaRS belongs to one of the most common, simple protein folds (the Rossmann fold) whereas Class II enzymes adopt a more rare and complex fold, might be used as argument for the primacy of Class I”.

Answer: Indeed, we agree that aaRSs associated with class I and II aaRSs evolved at a time when some proteins and canonical folds were already well-established. These ancient proteins might be associated with coenzyme biosynthesis enzymes such as biotin synthetase, or with biosynthesis of fatty acid derivatives, for example. We previously showed that all structural domains of biotin synthetase have structural homologs in multi-domain β-subunit of PheRS. Remarkable similarity when all structural domains of one multi-domain protein appear to be constituents of the other multidomain protein supports a concept of a common ancestor for two different enzymes [1,2].

In our discussion, we entertain the proposal that class II aaRSs were the first to replace ribozyme-based aminoacylation. Considerable evidence exists in favor of this point: 1) the amino acids formed in the Miller experiment are mostly class II-related; 2) the reconstructed chronology of amino acids introduction into the genetic code, as presented by E. Trifonov [3], strongly suggests the association of early amino acids with class II; 3) the so-called, more ancient “second genetic code”, located in the stem-loop of the tRNA, is primarily recognized by class II aaRSs; 4) our findings [4] suggest that organization of amino acid biosynthetic pathways, and clustering of aaRSs into different classes are intimately related to one another. A plausible explanation for such a relationship is dictated by early link between aaRSs and amino acid biosynthetic proteins. The aaRSs’ catalytic cores are highly relevant to the ancient metabolic reactions, namely to amino acid and cofactors biosynthesis. In particular it has been shown that class II aaRSs mostly associated with the primordial amino acids, while class I aaRSs are usually related to amino acids that evolved at a later stage [4]. The statement regarding the simplicity and structural priority of the Rossmann fold, at least, is non-trivial. For example it was hypothesized that aaRSs of two classes were originally associated with one common tRNA molecule [5,6]. Additionally, Carter and Duax in 2002 reported that complementary fragments of the specific DNA region in *Achlya klebsiana* code for proteins (not aaRSs) that have the same folds as class I and II synthetases [7].

1) Artymiuk PJ, Rice DW, Poirette AR, Willet P: **A tale of two synthetases**. *Nature Struct. Biol.* 1994, **1**:758-760.

2) Safro MG, Mosyak, L: **Structural similarities in the noncatalytic domains of phenylalanyl-tRNA and biotin synthetases**. *Protein Science*. 1995, **4**:2429-2432.

3) Trifonov EN: **Consensus temporal order of amino acids and evolution of the triplet code**. *Gene*. 2000, **261**:139-51.

4) Klipcan L, Safro M: **Amino acid biogenesis, evolution of the genetic code and aminoacyl tRNA synthetases**. *J. Theor Biol*. 2004, **238**:389-396.

5) Rodin S, Rodin A, Ohno S: **The presence of codon-anticodon pairs in the acceptor stem of tRNAs. ***Proc Natl Acad Sci U S A*. 1996, **93**:4537-42.

6) Ribas de Pouplana L, Schimmel P**: Aminoacyl-tRNA synthetases: potential markers of genetic code development. ***Trends Biochem Sci*., 2001, **26**: 591-6.

7) Carter CW, Duax WL: **Did tRNA synthetase classes arise on opposite strands of the same gene? ***Mol Cell.* 2002*,***10**:705–708.

b. Reviewer’s comment: “…I find the discussion of cysteine incorporation in Archaea (which the authors consider to be ancestral without any good reason) as well as the mechanism of PheRS to be largely irrelevant and more confusing than enlightening”.

Answer: Genome-wide analysis revealed that cysteine content was dramatically increased (a so- called “gainer” amino acid) only after the existence of LUCA, suggesting late incorporation of cysteine into the genetic code [8,9]. Indeed, in organisms using the standard CysRS-tRNA^Cys^ pathway of aminoacylation, this probably is the case. However, our concept is that high cysteine content in methanogenic Archaea (considered being very ancient) and the way it forms Cys-tRNA^Cys^ (via a tRNA-dependent pathway of cysteine biosynthesis, using L-phosphoserine as a precursor, and the class II aaRS SepRS [10]), suggests that cysteine was abundant in some ancient organisms. We used this example as an indication of the antiquity of tRNA-dependent pathways in such organisms. This point of view was supported very recently by results published by Zhang et. al. [11]: “These results indicate that tRNA-dependent Cys biosynthesis appeared 500 million years earlier (~3.5 Ga) than the tRNA-independent counterparts (~3.0 Ga), supporting a previous opinion that tRNA-dependent Cys biosynthesis had a very ancient origin (Klipcan et al., 2008)”.

8) Jordan IK, Kondrashov FA, Adzhubei IA, Wolf YI, Koonin EV, Kondrashov AS, Sunyaev S. **A universal trend of amino acid gain and loss in protein evolution**. *Nature*. 2005, **433**:633-8.

9) Brooks DJ, Fresco JR. **Increased frequency of cysteine, tyrosine, and phenylalanine residues since the last universal ancestor**. *Mol Cell Proteomics*. 2002, **1**:125-31.

10) Sauerwald A, Zhu W, Major TA, Roy H, Palioura S, Jahn D., Whitman WB, Yates JR 3rd, Ibba M, and Soll D. **RNA-dependent cysteine biosynthesis in archaea**. *Science*. 2005. **307**:1969-1972.

11) Zhang H-Y, Qin T, Jiang Y-Y, Caetano-Anollés G. **Structural phylogenomics uncovers the early and concurrent origins of cysteine biosynthesis and iron-sulfur proteins**. *J. Biom. Str. Dynamics*, 2012; **30**:542–545.

c) Reviewer’s comment: “…This is extremely unlikely to be the case because both classes of the aaRS seem to be rather late arrivals in the evolution of the respective protein folds, their emergence being antedated by extensive protein evolution [12,13]. The same holds for EF-Tu which belongs to a small branch within the huge GTPase superfamily [14].

Answer: We completely agree with the reviewer’s remark that the RNA based translation system was already well-established; thus, could be active without EF-Tu [15,16] and based on aaRSs ribozymes.

12) Aravind L, Mazumder R, Vasudevan S, Koonin EV: **Trends in protein evolution inferred from sequence and structure analysis**. *Curr Opin Struct Biol*. 2002, **12**:392-399.

13) Aravind L, Anantharaman V, Koonin EV: **Monophyly of class I aminoacyl tRNA synthetase, USPA, ETFP, photolyase, and PP-ATPase nucleotide-binding domains: implications for protein evolution in the RNA**. *Proteins*. 2002, **48**:1-14.

14) Leipe DD, Wolf YI, Koonin EV, Aravind L: **Classification and evolution of P-loop GTPases and related ATPases. ***J Mol Biol*. 2002, **317**:41-72.

15) Gavrilova LP and Spirin AS: **Stimulation of "non-enzymic" translocation in ribosomes by p-chloromercuribenzoate**. *FEBS Lett*. 1971, **17**:324-326.

16) Gavrilova LP, Kostiashkina OE, Koteliansky VE, Rutkevich NM and Spirin AS: **Factor-free (“Non-enzymic”) and factor-dependent systems of translation of polyuridylic acid by *Escherichia coli *ribosomes**. *J Mol Biol*. 1976. **101**:537-552.

This referee made remarks regarding the grammatical imprecision, misspellings, and quality of the written English. We accepted all his suggestions, and made corrections accordingly. The manuscript was extensively edited by a scientific editor.

## Abbreviations

aaRSs: aminoacyl-tRNA synthetases; PTC: Peptidyl transferase center; LUCA: Last universal common ancestor; aa-tRNAs: aminoacyl-tRNAs; aa-AMP: aminoacyl-adenylate; aa: amino acid; CysRS: Cysteinyl-tRNA synthetase; SepRS: *O*-phosphoseryl-tRNA synthetase; SepCysC: Cys-tRNA synthase; mitPheRS: mitochondrial PheRS.

## Competing interest

The authors declare that they have no competing interests.

## Authors’ contributions

MS and LK formulated hypothesis and wrote the manuscript. Both authors read and approved the final manuscript.
